# Osteomyelitis of the femur mimicking bone tumors: a review of 10 cases

**DOI:** 10.1186/1477-7819-11-283

**Published:** 2013-10-22

**Authors:** Po-Yen Huang, Po-Kuei Wu, Cheng-Fong Chen, Fang-Tsai Lee, Hung-Ta Wu, Chien-Lin Liu, Tain-Hsiung Chen, Wei-Ming Chen

**Affiliations:** 1 Department of Orthopaedics and Traumatology, Taipei Veterans General Hospital, 201, Section 2, Shi-Pai Road, Taipei 112, Taiwan; 2 Department of Orthopaedics and Traumatology, Kuang Tien General Hospital, 117, Shatian Road, Taichung 433, Taiwan; 3 Department of Radiology, Taipei Veterans General Hospital, 201, Section 2, Shi-Pai Road, Taipei 112, Taiwan; 4 Orthopaedic Department School of Medicine, National Yang- Ming University, 155, Section 2, Linong Street, Taipei 112, Taiwan

**Keywords:** Osteomyelitis, Mimicking bone tumor, *Klebsiella pneumoniae*

## Abstract

**Background:**

The clinical symptoms and radiographic appearance of osteomyelitis can mimic those of bone tumors.

**Methods:**

We reviewed 10 patients with osteomyelitis of the femur who were initially diagnosed as having bone tumors and were subsequently transferred to our institution.

**Results:**

Nocturnal pain of moderate intensity occurred in seven patients, and all 10 patients had elevated C-reactive protein levels. The radiographic findings included the following: a permeative, moth-eaten osteolytic lesion in six patients, an osteolytic lesion with sclerotic borders in three patients, and cortical destruction with pathological fracture in one patient. Magnetic resonance imaging was performed for eight patients, and only one had a positive penumbra sign. All patients underwent a surgical biopsy to confirm the final diagnosis for histological analysis and cultures. *Klebsiella pneumoniae* was detected in six patients and *Staphylococcus aureus*, the most common organism in osteomyelitis, was detected in three. Recurrence of infection occurred in five patients following debridement surgery; of these three had a *Klebsiella pneumoniae* infection. All patients received antibiotic treatment for an average of 20.4 weeks (range, 4 to 44) and surgical treatment an average of 1.8 times (range, 1 to 4). At the final follow-up, all patients were fully recovered with no signs of infection.

**Conclusions:**

When used in combination, clinical examinations, laboratory data, and radiographic findings can reliably distinguishing osteomyelitis from bone tumors.

## Background

Bone infection in the adult population is more likely to be exogenous rather than hematogenous in origin. This is partially because of the predilection for bacterial seeding of the bone ceases with closure of the epiphyses [1,2]. Therefore, hematogenous osteomyelitis is rare in individuals beyond their teens, occurring only in immunocompromised hosts. However, in the absence of trauma, systemic disease, or local infection, distinguishing between hematogenous osteomyelitis and a bone tumor is difficult [1,3].

Making an accurate clinical and radiographic diagnosis of bone lesions, such as soft-tissue swelling, cortical tunneling, focal cancellous lysis, focal cortical resorption, and a periosteal reaction remains a challenging endeavor. Magnetic resonance imaging (MRI), which has high sensitivity, is useful in the detection of pathologic changes in bone marrow and can provide precise information about the localization and extent of an infection, but not about any specific findings for osteomyelitis [1,3]. The penumbra sign is described as a thin layer of granulation tissue lining the abscess cavity in subacute myelitis, which has a higher intensity T1 signal than the cavity itself. The description of the penumbra sign was first published in 1998 by a group from the United Kingdom [4], and was thought to be helpful in differentiating between infection and neoplasm [5-8]. In one study, diagnoses made using the penumbra sign had a sensitivity and specificity of 70% and 99%, respectively [1,9].

However, it is still difficult to differentiate osteomyelitis from bone tumors, especially in long bones. Often the diagnosis can only be made after carefully studying the histologic features of the lesions [10].

The present series included patients who visited the Taipei Veterans General Hospital, whose diagnoses were questionable on clinical and radiographic grounds, and who later were proven to have inflammatory lesions of the bone on histologic analysis of biopsy specimens. We reviewed all patients who had osteomyelitis of the femur mimicking malignant bone tumors.

## Methods

We retrospectively reviewed the records of 10 patients (seven men and three women) who were diagnosed with osteomyelitis of the femur and treated at our institution between 2003 and 2011. The demographic data of all 10 cases are shown in Table 1. The mean age at the time of diagnosis was 37.9 years (range; 13 to 57 years).

**Table 1:** Date for the 10 cases

**Case**	**Age/sex**	**Nocturnal pain/BW loss**	**Delayed diagnosis (month)**	**Radiography**	**MRI**	**Preoperative diagnosis**	**Underlying disease**	**Treatment (antibiotics/weeks)**	**WBC/mm**^**3**^	**ESR (mm/h)/ CRP (mg/L)**	**Recurrence/pathological fracture/surgery times**	**culture**
1	57/M	+/-	4	Moth-eaten osteolysis		Ewing sarcoma		S+D+C (36)	10500	132/12.7	+/+/4	*Klebsiella pneumoniae*
				Laminated periosteal reaction								
2	33/M	+/+	6	Moth-eaten osteolysis		Ewing sarcoma	Drug abuser	S+D+C+O (40)	19900	Nil/15.4	+/+/4	*Klebsiella pneumoniae*
				Laminated periosteal reaction								
3	54/F	+/-	3	Moth-eaten osteolysis	Cortex destruction Soft tissue mass	Ewing sarcoma	DM	S+D+C+O (9)	10400	Nil/7.29	-/-/1	*Klebsiella pneumoniae*
				Laminated periosteal reaction								
4	37/F	-/-	1	Moth-eaten osteolysis	Cortex destruction	Leukemia	DM	S+D+C+O (4)	16500	91/12.9	-/-/1	*Klebsiella pneumoniae*
				Monolayer periosteal reaction	Penumbra sign							
5	37/M	-/-	8	Moth-eaten osteolysis Laminated periosteal reaction		Ewing sarcoma	DM	S+D+C+O (44)	16600	Nil/21.6	+/+/2	*Klebsiella pneumoniae*
6	14/M	+/ +	4	Osteolysis Laminated periosteal reaction	Extensive soft tissue	Ewing sarcoma		Casting (15)	7000	45/0.95	+/+/1	*Staphylococcus aureus*
7	13/M	-/-	1	Hyperostosis Septated periosteal reaction	Extensive soft tissue	Malignant change of osteomyelitis	DM	S+D+C (4)	12200	68/13	-/-/1	*Klebsiella pneumoniae*
8	30/M	+/-	4	Osteolysis	Soft tissue mass Cortex destruction	Metastatic lesion		S+D+C (6)	11000	58/1.13	-/-/ 1	*Staphylococcus aureus*
				Laminated periosteal reaction								
9	50/F	+/+	5	Osteolysis	Periosteal reaction			S+D+C (42)	10800	93/13.7	+/-/2	*Staphylococcus aureus*
				Laminated periosteal reaction								
10	54/F	+/-	3	Hyperostosis	Enhancing lesion	Metastatic tumors		S+D+C (4)	8700	NIL/1.15	-/-/1	*Propionibacterium*
				Laminated periosteal reaction								
Summary		Night pain: 7	AVG: 3.9				DM: 4	AVG: 20.4 weeks	Elevated: 8	Elevated CRP (>0.5 mg/L): 10	Recurrence/fracture: 5/4	
		Weight loss: 3					Drug abuser: 1					

BW, body weight; CRP, C-reactive protein; DM, diabetes mellitus; ESR, erythrocyte sedimentation rate; MRI, magnetic resonance imaging; S+D+C+O, sequestrectomy + debridement + bone cement + open reduction internal fixation; WBC, white blood cell.

**Figure 1:**
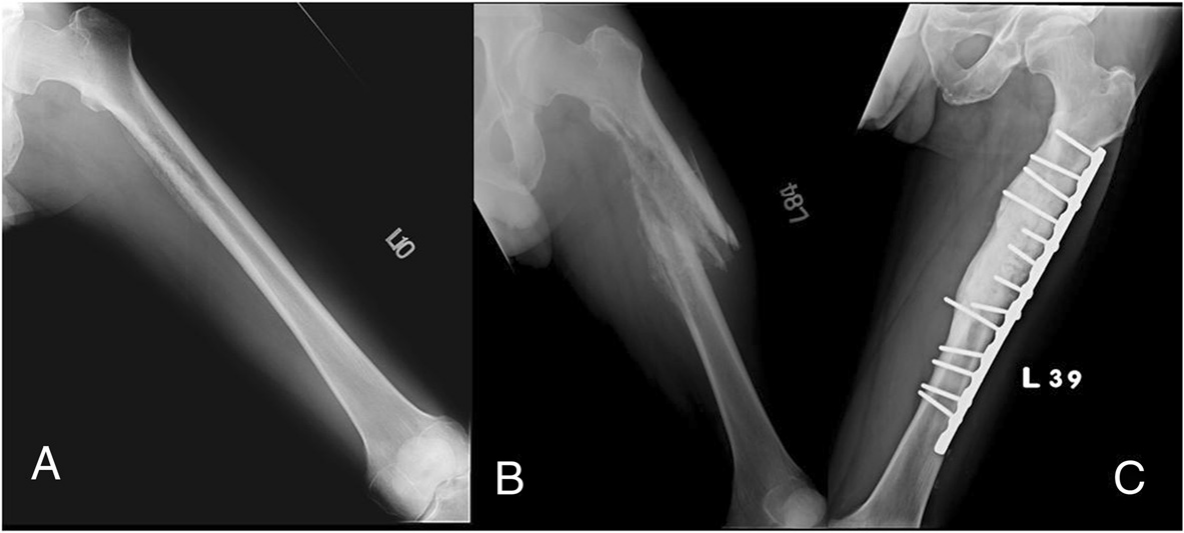
**A 57-year-old man who complained of nocturnal thigh pain for four months. (A)** Preoperative plain film showing a permeative osteolytic lesion and cortical disruption with laminated periosteal reaction. **(B)** Plain film taken one month after debridement surgery, showing a pathological fracture with severe periosteal reaction and cortex thinning. **(C)** Two years after open reduction and internal fixation (ORIF) and multiple debridement surgeries: antibiotic impregnated-bone cement at medial cortex and bony union without recurrent signs.

**Figure 2:**
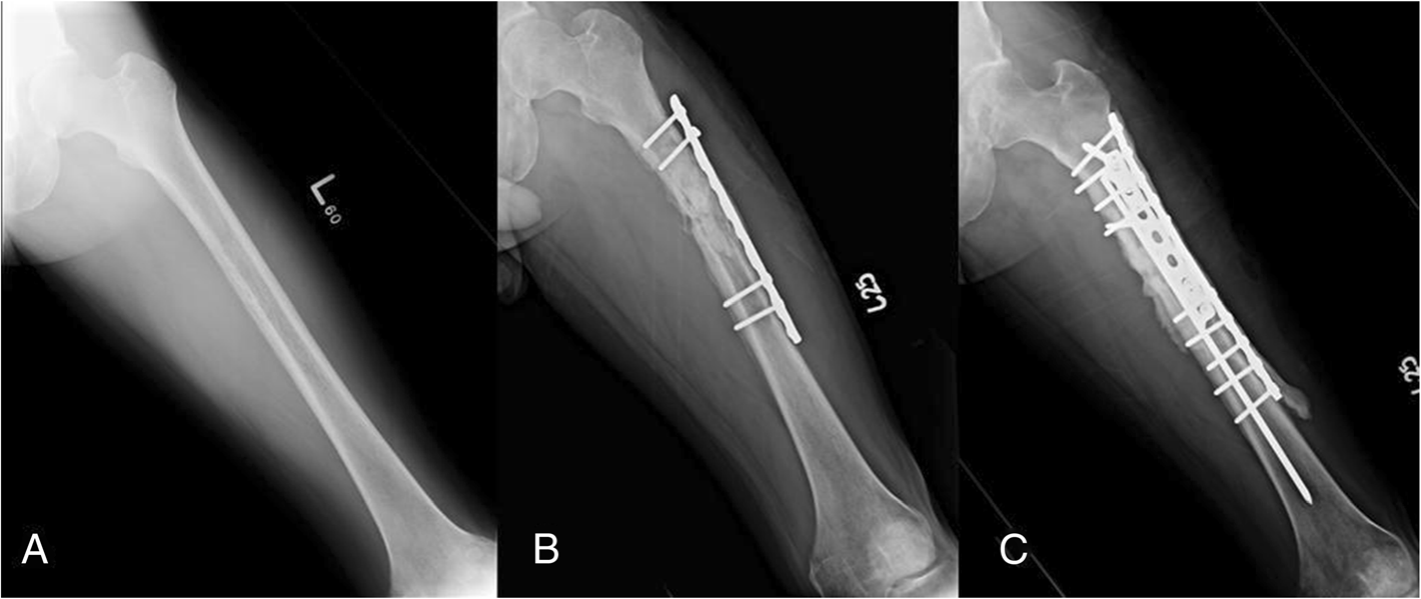
**A 33-year-old man who complained of nocturnal thigh pain and weight loss noted over six months. (A)** A plain film of the femur showing moth-eaten osteolytic lesion with cortical destruction and laminated periosteal reaction. **(B)** A plain film taken six weeks after prophylactic fixation and debridement surgery showing recurrent osteomyelitis and progressive destruction of the medial cortex with pathological fracture. **(C)** A plain film taken one year after the final surgery showing bony healing without recurrence.

**Figure 3:**
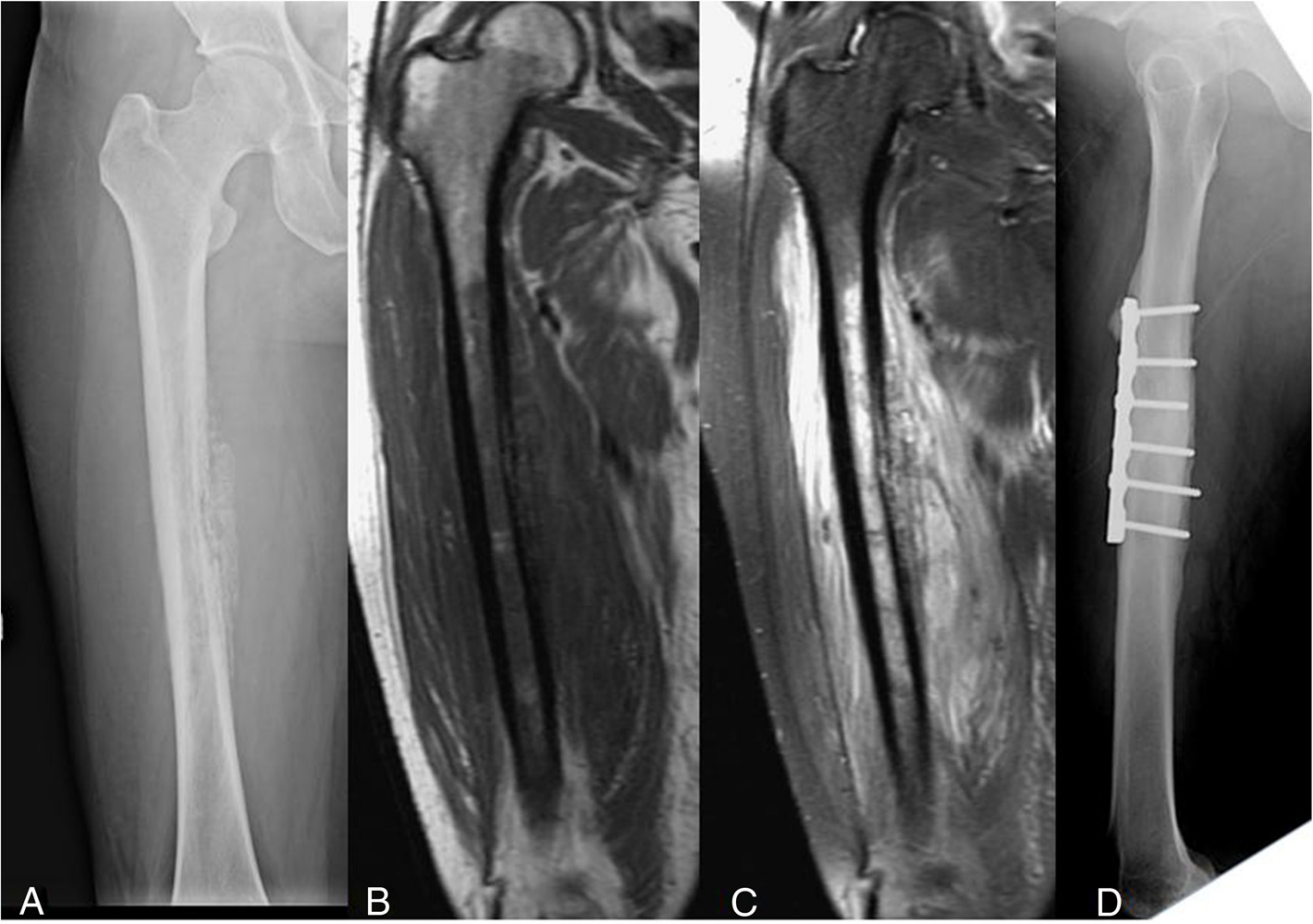
**A 54-year-old woman with a history of type 2 diabetes mellitus who complained of moderate nocturnal thigh pain for three months. (A)** A plain film of the femur showing a laminated periosteal reaction with partial cortical destruction and moth-eaten osteolytic lesions in the medulla. **(B**, **C)** T1- and T2-weighted magnetic resonance imaging (MRI) showing cortical destruction with extensive soft tissue edema. **(D)** A plain film taken 18 months after debridement and open reduction and internal fixation (ORIF) surgery showing bony healing with no signs of recurrence.

**Figure 4:**
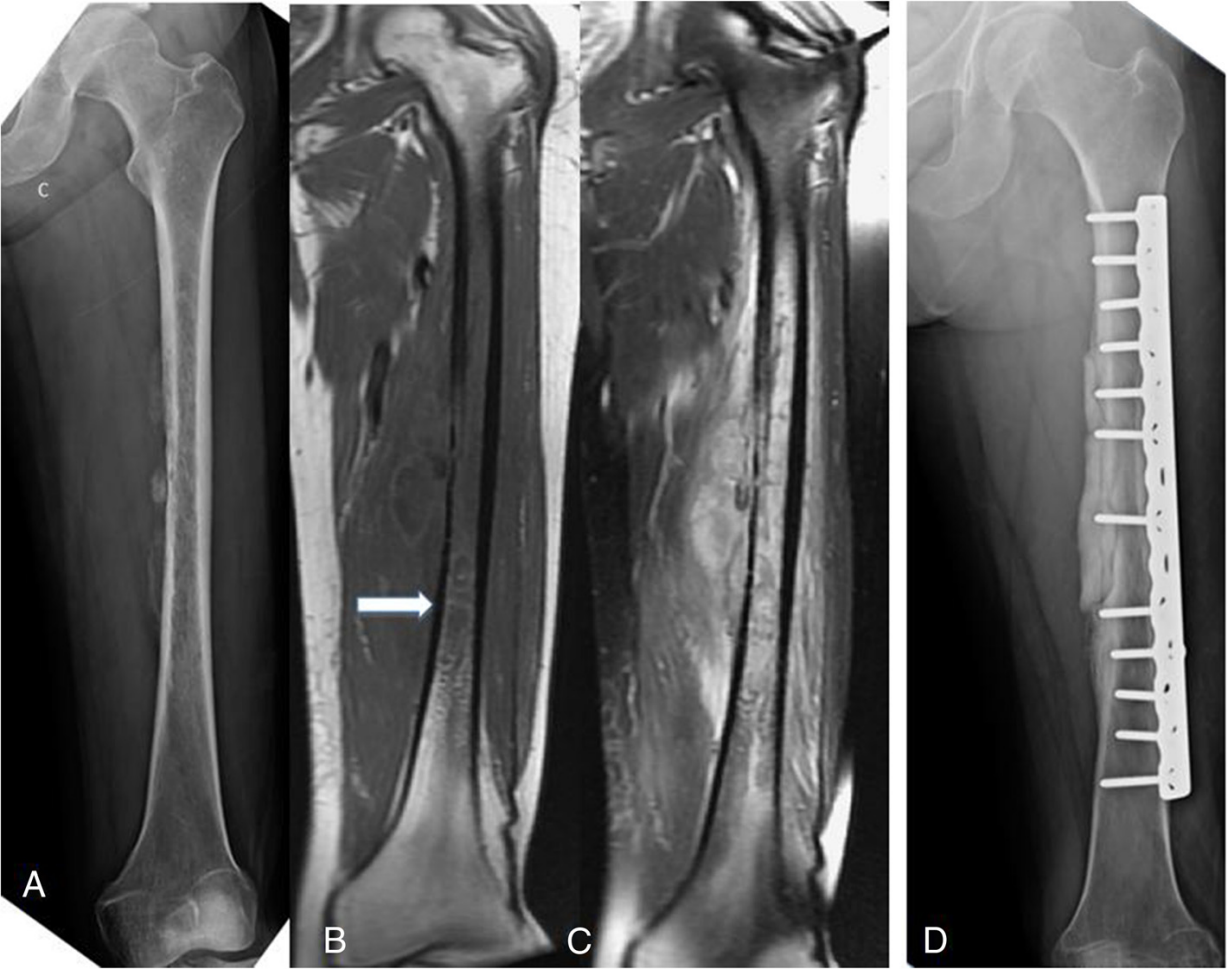
**A 37-year-old woman with type 2 diabetes mellitus who complained of painful swelling of the thigh for one month. (A)** A plain film showing a thin monolayer periosteal reaction with cortical destruction and moth-eaten osteolytic lesion. **(B, C)** Unenhanced T1-weighted magnetic resonance imaging (MRI) showing cystic lesion with a hyperintense granulation layer surrounding the central abscess cavity, and a T2-weighted image showing extensive soft tissue edema and lobulated abscess accumulation, representing the penumbra sign. **(D)** A plain film taken one year after the debridement and prophylactic ORIF surgery showing bony union and bone cement at the medial wall without signs of recurrence.

**Figure 5:**
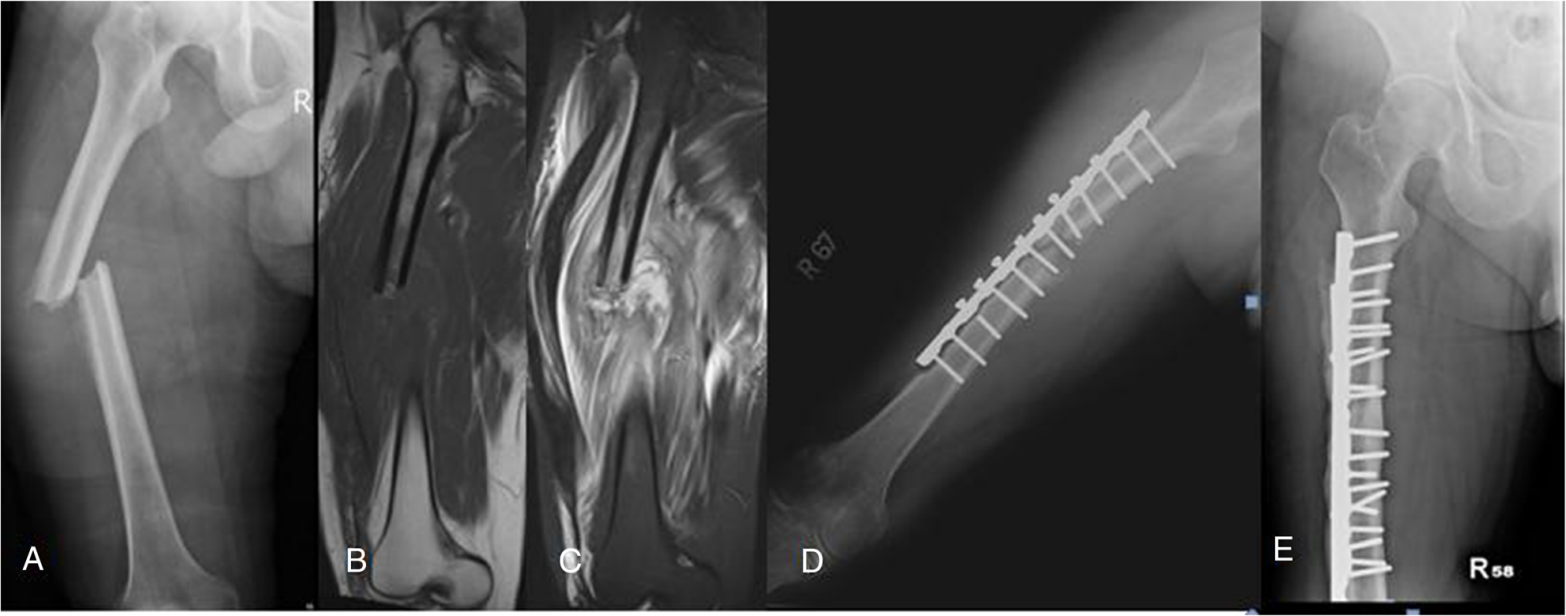
**A 37-year-old man with a history of type 2 diabetes mellitus. (A)** Plain film of the femur showing a pathological fracture with lateral cortex thinning and laminated periosteal reaction. **(B, C)** T1- and T2-weighted magnetic resonance imaging (MRI) showing severe soft tissue swelling and edematous change. **(D)** Plain film taken two months after open reduction and internal fixation (ORIF) and debridement surgery showing recurrent osteomyelitis with loss of reduction and laminated periosteal reaction of the medial cortex. **(E)** Plain film taken one year after revision debridement and ORIF surgery showing bony union without signs of osteomyelitis recurrence.

**Figure 6:**
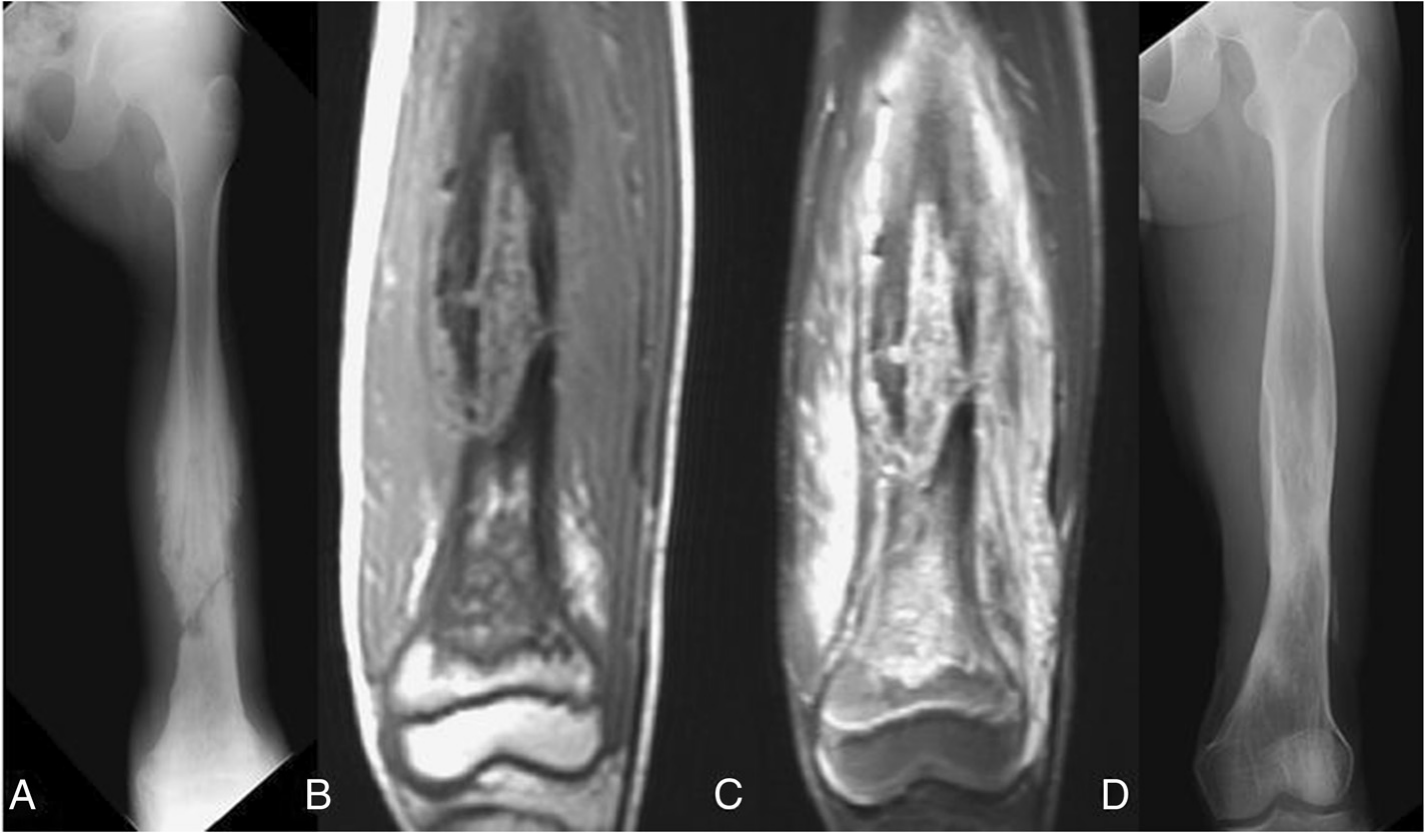
**A 14-year-old boy who experienced moderate progressive thigh pain, especially at night, for four months. (A)** A plain film of the femur showing a laminated periosteal reaction with pathological fracture at the distal femur. **(B**, **C)** Magnetic resonance imaging (MRI) showing tumor-like lesion at the marrow surrounded with extensive soft tissue edema. **(D)** A plain film of the femur taken two years after casting and debridement surgery showing bony union.

All 10 cases were referred to our orthopedic oncology service because of a strong initial suspicion of a malignant bone, with no clear differentiation made based on clinical and radiographic findings. The final diagnosis of osteomyelitis was made based on histological and microbiological findings. All patients received a combination of surgical treatments, including sequestrectomy, debridement, and placement of an antibiotic-impregnated polymethylmethacrylate (PMMA) bone cement. Fixation with a broad dynamic compression plate was performed in cases with pathological fractures or severe cortical destruction. Three of the ten patients with severe osteolytic lesions that had predisposed them to femoral fractures (Figures 1A, 2A, 3A, and 4A) underwent prophylactic internal fixation with a broad dynamic compression plate (DCP). Two of the ten patients initially presented with a pathological femoral shaft fracture: one underwent open reduction and internal fixation (ORIF) (Figure 5), and the other was treated with casting (Figure 6). The following data were collected and analyzed: gender; age; intervals between the onset of symptoms and surgical treatment; clinical symptoms; laboratory data; imaging studies, including plain film and magnetic resonance imaging; microbiological findings, surgical treatment times; and antibiotic treatment period. This study was approved by the Institutional Review Board of our hospital. The requirements for informed consent were waived because of the retrospective nature of this study.

## Results

We reviewed the medical records of each patient to identify the clinical symptoms, laboratory data, radiographic appearance, and treatment. The mean interval between the onset of symptoms and surgical treatment was four months (range; 4 weeks to 8 months). Seven of ten (70%) patients complained of nocturnal thigh pain of moderate intensity, and two patients (70%) experienced weight loss.

### Laboratory data

All 10 patients had elevated C-reactive protein (CRP) levels (>0.5 mg/dL), and six had elevated erythrocyte sedimentation rates (ESRs) of >30 mm/h (range, 45 to 132). Four of ten patients had leukocytosis (white blood cell (WBC) count >11,000/mm^3^).

### Radiographic findings

**Figure 7:**
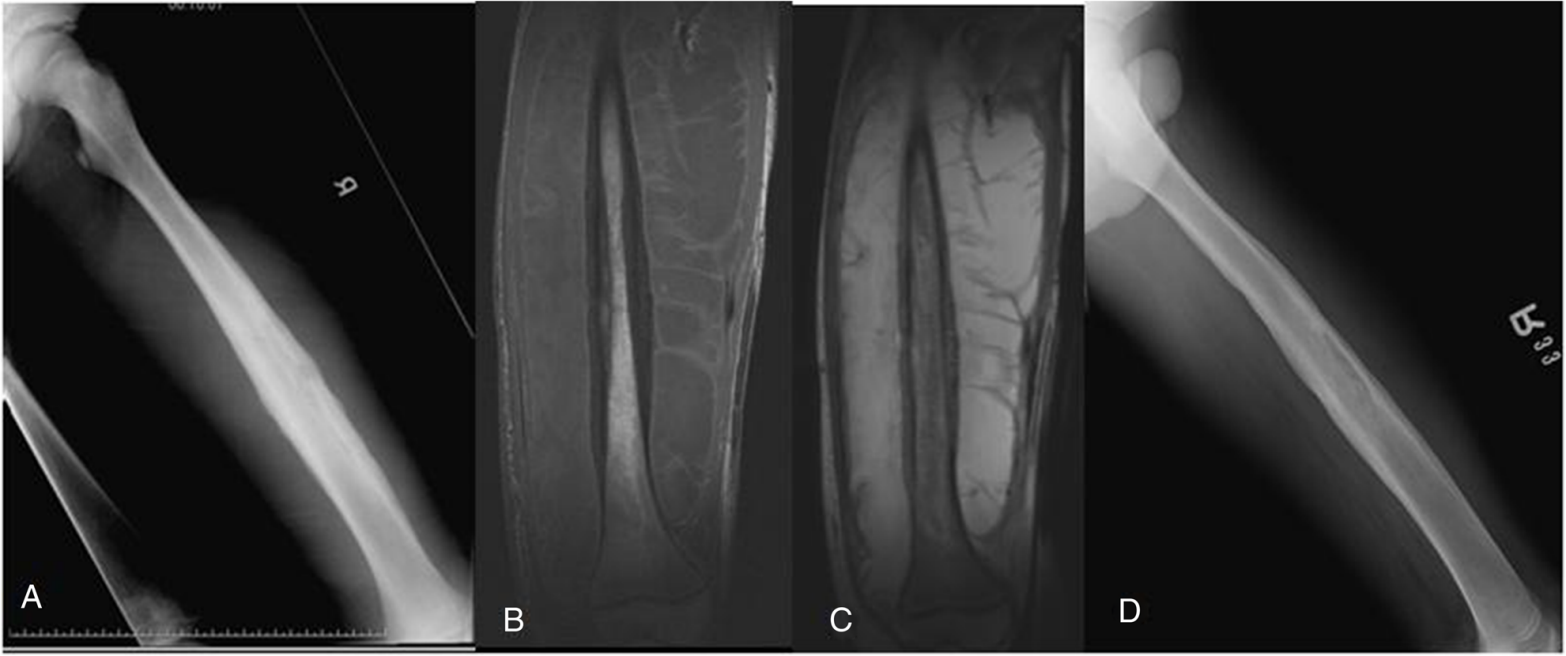
**A 13-year-old boy who complained of right thigh pain for one month. (A)** Plain film of the femur showing a sclerotic border with septated periosteal reaction. **(B, C)** T1 and T2-weighted magnetic resonance imaging (MRI) showing septated periosteal reaction with extensive soft tissue swelling. **(D)** Plain film radiograph taken one year after debridement surgery.

Plain film radiographs of the femur showed the following: osteolysis with a sclerotic border in two patients, a central transparent zone of moth-eaten osteolysis in six patients, an osteolytic pattern with pathological fracture in one patient (Figure 5A), a laminated periosteal reaction in six patients (60%), monolayer in three patients, and septation in one patient (Figure 7A).

**Figure 8:**
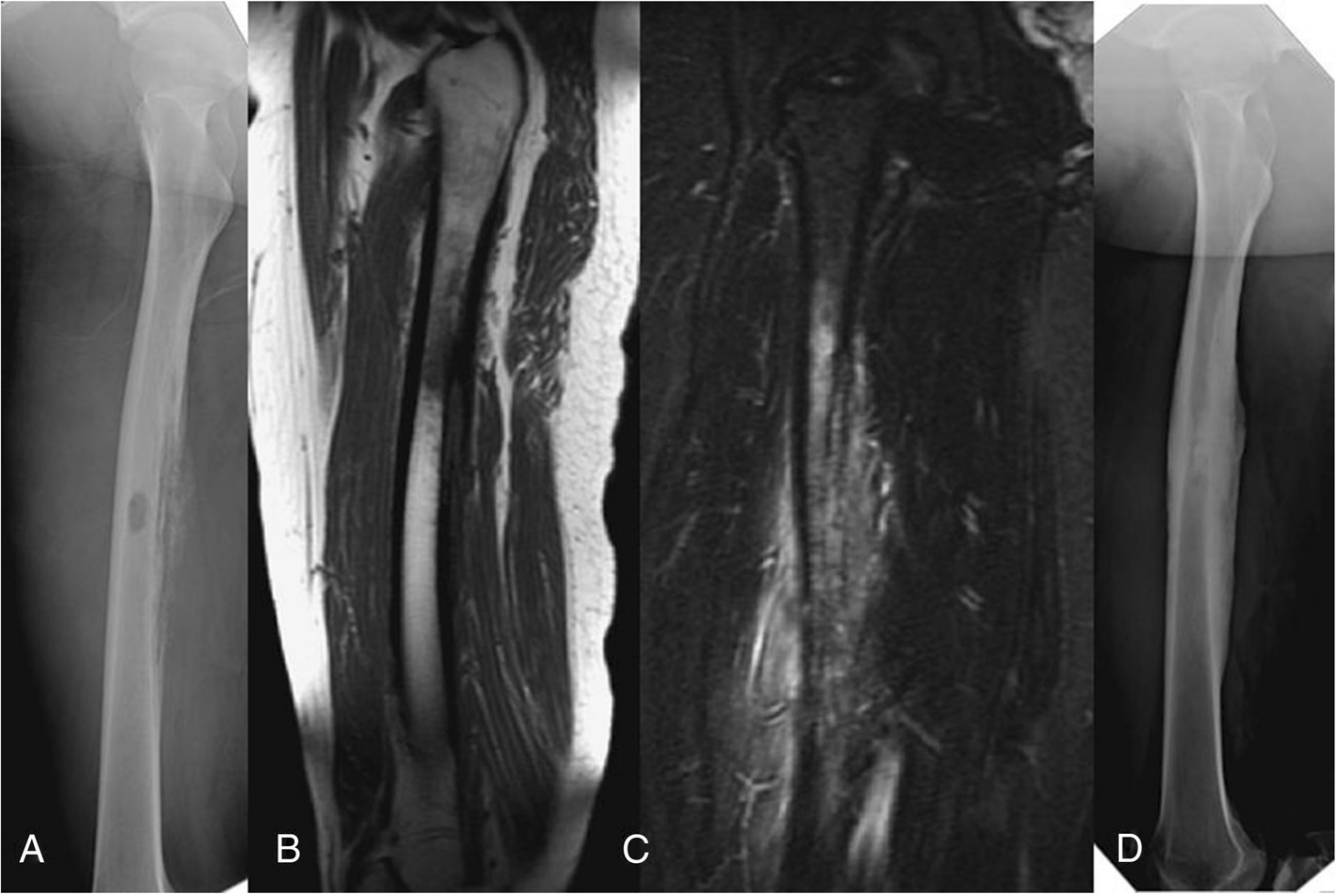
**A 30-year-old healthy man who complained of moderate nocturnal thigh pain for four months after he underwent a bone biopsy. (A)** A plain film of the femur showing laminated periosteal reaction with cortical destruction. **(B, C)** Magnetic resonance imaging (MRI) showing cortical destruction with bone marrow and soft tissue edematous change. **(D)** A plain film taken one year after the surgery showing bony union without recurrence.

MRI evaluation showed cortical destruction with soft tissue mass or abscess accumulation and edematous changes. The penumbra sign was found in only one of our patients (Figure 4B). Osteomyelitis mimicking Ewing sarcoma was found in five (Figures 1A, 2A, 3A, 5A, 6A) patients and that mimicking metastatic lesions were found in two patients (Figure 8A).

### Treatment

A comprehensive preoperative survey was performed, including the Mirels criteria to evaluate the possibility of pathological fracture [11]. All patients received an open biopsy with an intraoperative frozen section. First-generation cephalosporin was used prophylactically during surgery. If frozen section analysis was negative for malignancy, we performed curettage and debridement of the abscess and necrotic bone, combined with internal fixation with a broad DCP in the cases with pathological fracture, or impending pathological fracture (in cases whose Mirels score was >8), with the exception of case 2 (Figure 1A), who received debridement surgery alone, and had a subsequent fracture two months postoperatively (Figure 1B). After debridement and sequestrectomy, the vancomycin-impregnated PMMA bone cement was placed around the lesion and the plate for controlling local infection (Figure 1C). The infected and necrotic tissue was sent for permanent formalin-fixed pathological analysis and culture.

### Pathogen

All 10 cases were positive for pathogens. *Klebsiella pneumoniae* was isolated in six (60%) patients, *Staphylococcus aureus* in three (30%) patients, and *Propionibacterium* in one (10%) patient. Five of the six patients with *Klebsiella pneumoniae* were male, while three of six (50%) had preoperative history of nocturnal pain, and one had experienced weight loss. Four of the six (66%) patients had a past medical history of type 2 diabetes mellitus, while one had a history of intravenous drug abuse. All six had elevated CRP levels. Additionally radiography showed moth-eaten osteolysis with a laminated periosteal reaction in five of six (83.3%) patients. Three of six (50%) patients had recurrent osteomyelitis and underwent multiple surgeries and prolonged antibiotic treatment.

### Follow-up

All patients were followed up regularly at our outpatient clinic. Recurrent osteomyelitis following the primary surgery was noted in five patients, all of whom underwent a second debridement procedure, ORIF surgery, and prolonged antibiotic treatment. Four of the five had a history of pathological fracture, including one patient who initially presented with a fracture, and three who experienced postsurgical fractures. All patients received intravenous antibiotics treatment after primary surgery, and took oral antibiotics after they were discharged. Antibiotic treatment was continued until clinical symptoms improved and CRP levels decreased to within the normal range (<0.5 mg/L). Antibiotic treatment continued for an average of 20.4 weeks (range; 4 to 44 weeks). At the most recent follow-ups, no recurrences of infection were identified based on clinical symptoms and radiographic results. The average interval of antibiotic treatment was 35.4 weeks (range; 15 to 44 weeks) for patients with recurrence of infection, and 5.4 weeks (range; 4 to 9 weeks) for those without recurrence. The average number of surgical treatments for patients in the recurrence group was 2.6 (range; 2 to 4).

## Discussion

Osteomyelitis is difficult to differentiate from malignant bone tumors, as it lacks specific signs and symptoms. Distinguishing between the two potential diagnoses is important in order to initiate proper clinical management.

Shimose *et al*. [1] reported on 244 cases that were tentatively diagnosed as malignant bone tumor based on imaging results; however, 15 of these cases were osteomyelitis. The clinical symptom of pain was noted in all osteomyelitis cases, and swelling of the limb was present in eight cases. In this study, laboratory data showed elevated CRP levels in nine (60%) patients and leukocytosis in three (20%). Radiographs of the osteomyelitis cases showed osteolysis in 12 (80%) patients. Pathogens were found in 11 of 15 patients, including *Staphylococcus aureus* in eight patients, *Salmonella* in two patients, and *Staphylococcus epidermidis* in one patient.

Cottias *et al*. [12] reported on 21 osteomyelitis cases mimicking bone tumor. Pain was also noted in all cases, but only half complained of nocturnal pain. Two of twenty-one (9%) patients had leukocytosis, and 30% had elevated ESRs.

Nocturnal pain was frequently misdiagnosed as a bone tumor in our cases (70%). Weight loss was found in only 20% of our cases.

In our cases, WBC count was inconsistent with that reported previously, but ESRs and CRP levels were consistent with previous reports indicating high levels are relatively sensitive indicators for distinguishing osteomyelitis from bone tumors, but not from metastatic tumors and Ewing sarcoma [1,3]. Only 40% of patients had leukocytosis, and this may not have been indicative of osteomyelitis. However, other studies have shown that laboratory investigations were inconsistent and did not aid in diagnosing subacute osteomyelitis [1,2,13]. In our series, there was an average 3.9 months (range; 1 to 8) delay in diagnosis, which may explain the constant elevation in CRP levels and ESRs in our patients.

Although radiographic analysis is important, making a distinction between the two conditions is difficult. The radiographic appearances of osteomyelitis are well documented, but can often be mistaken for various benign and malignant bone tumors [5]. The most common radiographic appearance in our series was a permeative moth-eaten osteolytic lesion with partial cortical destruction, which was found in seven patients, a cortical sclerotic rim surrounding the zone found in two patients, and osteolysis with pathological fracture in one patient. All lesions may mimic malignant bone tumors, thus making diagnosis more difficult based on radiographic findings alone.

MRI is sometimes helpful in diagnosing osteomyelitis. However, MRI findings have been reported as nonspecific and variable depending on the anatomic location, cause, and duration [9]. Grey *et al*. [3,4] reported on the significance of the penumbra sign on T1-weighted images in subacute osteomyelitis. They defined the penumbra sign as a transitional zone with relatively high signal intensity located between the abscess and sclerotic bone marrow on unenhanced T1-weighted images. The penumbra is isointense relative to muscle on T1-weighted, enhances on contrast administration, and is hypointense on T2-weighted [4]. They identified the penumbra sign in 24 of 32 cases (75%) of subacute osteomyelitis in their studies. They concluded that the penumbra sign was a characteristic MRI finding of subacute osteomyelitis. Moreover, this sign was reported with high sensitivity and specificity to subacute osteomyelitis [1,4]. However the target appearance and the penumbra sign were observed on MRI in only one patient in our study (Figure 4B). The lower sensitivity of the penumbra sign in our series may be due to two reasons: first, most of our cases had diaphyseal lesions, while previous publications reported on cases with metaphyseal lesions, and second, our patients had severe osteomyelitis and therefore, had a higher percentage of cortical breakthrough instead of penumbra sign formation.

Most cases of hematogenous osteomyelitis are monomicrobial [6-8,14]. Different types of organisms can cause osteomyelitis, based on several risk factors. *Staphylococcus aureus* has been found to be the most common cause of infection in athletes [1,15]; *Pseudomonas aeruginosa* and *Serratia marcescens* are found more frequently in intravenous drug abusers [10,16]; and osteomyelitis caused by *Salmonella* and *Propionibacterium* has been well described in patients with sickle cell disease [11,17,18]. Even though osteomyelitis caused by *Klebsiella pneumoniae* has previously been observed in diabetes mellitus patients [1,19-21], very few case series have reported such a high percentage of femur osteomyelitis in this setting. We found that *Klebsiella pneumoniae* was isolated in 60% of bone-tumor-mimicking femur osteomyelitis patients. In a series of 101 patients with *Klebsiella* bacteremia, underlying diabetes was present in 36% of the patients [12,22]. Among our patients, four of six (66%) had the predisposing factor of type II diabetes mellitus, and one of six had a history of intravenous drug abuse.

Among the six cases infected with *Klebsiella pneumoniae*, 83% presented with moth-eaten osteolytic lesions and laminated periosteal reaction visible on radiographs, which mimicked Ewing sarcoma. Additionally, three of six (50%) had recurrent osteomyelitis with pathological fractures. A high probability of type II diabetes mellitus was found in four of the six cases, which is consistent with that found in previous reports. Overall, pathological fractures were found in five cases. Eighty percent of patients with pathological fractures had recurrent osteomyelitis that required a longer antibiotic treatment period and additional debridement surgeries.

### Limitations

Our study had several limitations. First, this was a retrospective case series study, rather than a prospective study. Second, the study population was relatively small. Third, all of the study subjects underwent open biopsy and surgery, without a control group for comparison. Nevertheless, osteomyelitis of the femur is rare, and osteomyelitis of the femur mimicking bone tumors is extremely rare. As this condition is difficult to diagnose, an open biopsy was required to obtain a definitive diagnosis in these cases.

## Conclusions

This review of 10 unusual cases of osteomyelitis of the femur mimicking bone tumors emphasizes the importance of clinical history, laboratory investigations, and radiographic interpretations in the diagnosis of this condition, which is generally difficult to detect. Moreover, it is difficult to distinguish between osteomyelitis of the femur and bone tumors by plain film radiography, and this condition commonly mimics Ewing sarcoma. In our series, accurate diagnosis of this condition was also difficult by using MRI, which is believed to be a sensitive and useful modality. The penumbra sign on MRI, which is reported to be highly specific for osteomyelitis, was detected in only one case in our series. However, elevated CRP levels and ESRs found to be consistent among the cases with this condition are relatively sensitive indicators for distinguishing osteomyelitis from bone tumors. But sometimes, Ewing sarcoma can present with similar laboratory data. Hence, we recommend that open biopsy should be performed in all cases for accurate diagnosis and for obtaining an adequate specimen for culture. Surprisingly, *Klebsiella pneumoniae* was the most commonly noted pathogen in our series. The relationship between *Klebsiella pneumoniae* and femur osteomyelitis mimicking malignant bone tumor requires further studies to be confirmed.

## Abbreviations

CRP: C-reactive protein; DCP: Dynamic compression plate; ESR: Erythrocyte sedimentation rate; MRI: Magnetic resonance imaging; ORIF: Open reduction and internal fixation; PMMA: Polymethylmethacrylate; WBC: White blood cell.

## Competing interests

The authors declare that they have no competing interests.

## Authors’ contributions

PYH participated in the acquisition of data and writing of the manuscript. PKW participated in the design of the study and revising the manuscript. CFC participated in the design of the study. FTL participated in the writing of the manuscript. HTW participated in the revising of the manuscript. CLL participated in the design of the study. THC and WMC participated in the direct clinical care (diagnosis, decision making, and treatment) of the patients and design of the study. All authors read and approved the final manuscript.
